# Regenerative Biomaterials and Emerging Biological Strategies for Peri-Implantitis: Current Clinical Evidence from a Structured Narrative Review

**DOI:** 10.3390/jfb17100505

**Published:** 2026-10-06

**Authors:** Jan Łoś, Małgorzata Zawrzykraj-Motylińska, Milena Deptuła-Knioła, Katarzyna Gurzawska-Comis, Barbara Kochańska, Michał Pikuła

**Affiliations:** 1 Laboratory of Tissue Engineering and Regenerative Medicine, Division of Embryology, Faculty of Medicine, Medical University of Gdańsk, 80-211 Gdańsk, Poland; milena.deptula@gumed.edu.pl; 2 Potokowa Center for Implantology and Facial Surgery, 80-283 Gdańsk, Poland; 3 Division of Clinical Anatomy, Faculty of Medicine, Medical University of Gdańsk, 80-211 Gdańsk, Poland; m.zawrzykraj@gumed.edu.pl; 4 Department of Dentistry and Oral Health, Aarhus University, Vennelyst Boulevard 9, 8000 Aarhus C, Denmark; kasia.gurzawska-comis@dent.au.dk; 5 Department of Conservative Dentistry, Medical University of Gdańsk, 80-210 Gdańsk, Poland; barbara.kochanska@gumed.edu.pl

**Keywords:** dental implant, peri-implantitis, implantological treatment, biomaterials, cell therapies

## Abstract

Peri-implantitis is a plaque-associated inflammatory condition characterized by progressive loss of supporting peri-implant bone and remains one of the most challenging biological complications in implant dentistry. Although reconstructive procedures are increasingly used, the relative effectiveness of different regenerative biomaterials and biologically enhanced strategies remains uncertain. This structured narrative review critically evaluates current clinical evidence for regenerative biomaterials used in peri-implantitis treatment and places emerging biological and cell-based approaches within their appropriate translational context. A structured literature search was conducted in PubMed/MEDLINE. Particular emphasis was placed on direct human evidence concerning enamel matrix derivative, porous titanium granules, deproteinized bovine bone mineral (DBBM), hydroxyapatite, biphasic calcium phosphate, and platelet-rich fibrin, while periodontal, endodontic, and preclinical studies were considered indirect or translational evidence where relevant. Available clinical studies indicate that reconstructive procedures can improve radiographic defect fill and selected peri-implant clinical parameters; however, radiographic improvement does not necessarily correspond to complete disease resolution or true re-osseointegration. Among currently evaluated grafting materials, DBBM, porous titanium granules, and hydroxyapatite are supported by direct human clinical evidence, although heterogeneity in HA formulations and accompanying treatment protocols limits material-specific conclusions. BCP-containing reconstructive protocols have also been evaluated clinically, but the independent contribution of BCP remains difficult to isolate. Enamel matrix derivative and platelet-rich fibrin may provide biologically active adjunctive effects, although their independent contribution within multimodal regenerative protocols remains difficult to determine. Emerging cell-based and cell-derived strategies offer additional regenerative potential but remain predominantly supported by preclinical or translational evidence. No single biomaterial has demonstrated universal superiority. Future studies require standardized disease definitions, defect classifications, outcome measures, adequately powered multicentre trials, long-term follow-up, and improved patient and defect stratification to determine which regenerative strategies provide clinically meaningful and durable benefits.

## 1. Introduction

Peri-implantitis is a plaque-associated pathological condition affecting the tissues surrounding dental implants, characterized by inflammation of the peri-implant mucosa and progressive loss of supporting bone [1,2]. If left untreated, the disease may lead to increasing peri-implant bone destruction, loss of implant stability, and ultimately implant failure [2,3]. Because successful management frequently requires the integration of periodontal, surgical, prosthetic, and maintenance-related considerations, peri-implantitis remains one of the most challenging biological complications in contemporary implant dentistry [4].

The development and progression of peri-implantitis are multifactorial. Microbial biofilm and the resulting host inflammatory response constitute central components of the disease process, while patient-related and local factors may modify susceptibility, severity, and progression [2,5]. A history of periodontitis, inadequate plaque control, smoking, systemic conditions, and irregular supportive care have been associated with an increased risk of peri-implant disease [5]. Local tissue conditions and prosthetic factors may further influence plaque accumulation, accessibility for oral hygiene, and the maintenance of peri-implant tissue health [4]. In contrast to natural teeth, dental implants lack a periodontal ligament and cementum, and the peri-implant mucosal barrier represents an important protective interface between the oral environment and the underlying implant-supporting tissues [6]. Disruption of this biological equilibrium may facilitate persistent inflammation and progressive tissue destruction.

The reported prevalence of peri-implantitis varies considerably among studies [7]. This variability reflects not only differences between patient populations but also substantial methodological heterogeneity. Different case definitions, thresholds for probing depth and radiographic bone loss, duration of implant function, follow-up periods, and the use of patient-level versus implant-level analyses can markedly influence prevalence estimates [7]. Consequently, apparently divergent prevalence rates should be interpreted in the context of the diagnostic criteria and methodological design used in individual studies rather than as directly comparable estimates of disease frequency.

Accurate diagnosis of peri-implantitis therefore requires a combined clinical and radiographic assessment. Contemporary diagnostic principles emphasize the presence of peri-implant inflammation, bleeding and/or suppuration on probing, increased probing depth compared with previous examinations when such information is available, and progressive bone loss beyond the initial physiological remodelling associated with implant placement and loading [1,2]. When previous clinical or radiographic records are unavailable, diagnosis requires careful interpretation of the current clinical and radiographic findings [1,3]. Importantly, no single probing-depth value or characteristic radiographic morphology should be considered independently diagnostic of peri-implantitis [1]. Crater-like or funnel-shaped bone defects may frequently accompany the disease but represent morphological manifestations of bone loss rather than standalone diagnostic criteria.

The identification of peri-implantitis should also include assessment of factors that may contribute to disease persistence or influence treatment prognosis. Plaque accumulation, oral hygiene, smoking, systemic health, previous periodontal disease, local soft-tissue conditions, implant position, prosthetic design, and accessibility for decontamination may all affect treatment planning and long-term outcomes [8]. Accordingly, successful management should not focus exclusively on reconstructing the resulting bone defect but should first aim to control inflammation and reduce modifiable risk factors.

Initial treatment commonly includes mechanical biofilm removal, improvement of oral hygiene, and appropriate control of local inflammatory conditions. Surgical intervention may subsequently be considered when non-surgical therapy fails to achieve adequate disease control or when the morphology and extent of the peri-implant defect require direct access and reconstruction [9]. Depending on the clinical situation, surgical treatment may involve resective, regenerative, or combined approaches. The decision to attempt regeneration should therefore not be based solely on the amount of bone loss but should also consider defect morphology, implant stability, accessibility for surface decontamination, local tissue conditions, prosthetic restorability, and patient-related factors [8,9,10,11].

Regenerative treatment of peri-implantitis is particularly challenging because successful reconstruction requires simultaneous control of infection, preservation or restoration of a stable regenerative space, and formation of new mineralized tissue around an implant surface previously exposed to inflammation. A variety of regenerative biomaterials have therefore been investigated, including deproteinized bovine bone mineral, hydroxyapatite, biphasic calcium phosphate, porous titanium granules, enamel matrix derivatives, barrier membranes, and biologically active adjuncts [12]. These materials differ substantially in their biological properties, resorption characteristics, mechanical stability, and level of clinical evidence.

Despite the increasing number of studies concerning regenerative treatment of peri-implantitis, the available evidence remains heterogeneous. Previous reviews have evaluated reconstructive procedures and individual biomaterials; however, direct comparison between regenerative strategies remains difficult because of differences in peri-implantitis definitions, defect morphology, decontamination protocols, surgical techniques, outcome measures, and follow-up periods [13]. Evidence derived from periodontal regeneration or preclinical tissue-engineering models may provide relevant mechanistic or translational context but should not be considered equivalent to direct clinical evidence from peri-implantitis treatment [14,15].

Therefore, the aim of the present structured narrative review was to critically evaluate currently available regenerative biomaterials used in the treatment of peri-implantitis, with particular emphasis on their biological properties, clinical effectiveness, limitations, and strength of supporting evidence. The review additionally discusses emerging biologically enhanced and cell-based regenerative strategies while clearly distinguishing direct clinical evidence in peri-implantitis from indirect periodontal, translational, and preclinical findings.

## 2. Aim and Methodology

The aim of this structured narrative review was to critically evaluate regenerative biomaterials currently used in the surgical treatment of peri-implantitis and to contextualize emerging biologically enhanced regenerative strategies. Particular emphasis was placed on the biological mechanisms, clinical effectiveness, limitations, and level of supporting evidence for bone substitute materials, enamel matrix derivatives, porous titanium granules, calcium phosphate-based materials, autologous platelet concentrates, and emerging cell-based approaches. A further objective was to distinguish interventions supported by direct clinical evidence in peri-implantitis from strategies supported predominantly by indirect periodontal, translational, or preclinical evidence.

A structured narrative approach was selected because the available literature is characterized by substantial heterogeneity in peri-implantitis case definitions, defect morphology, implant surface characteristics, decontamination protocols, regenerative materials, surgical techniques, outcome definitions, and follow-up periods. Accordingly, the purpose of this review was not to estimate a pooled treatment effect, but rather to critically compare the biological rationale, clinical outcomes, limitations, and translational relevance of different regenerative strategies. The preparation of this review was informed by the principles of the Scale for the Assessment of Narrative Review Articles (SANRA) [16].

### 2.1. Literature Search Strategy

A structured literature search was conducted in PubMed/MEDLINE from database inception to 14 July 2026. Material-specific searches were performed for enamel matrix derivative, porous titanium granules, deproteinized bovine bone mineral, hydroxyapatite, biphasic calcium phosphate, platelet-rich fibrin, and regenerative/reconstructive peri-implantitis therapy. The complete search strategies are provided in Supplementary File S1. Reference lists of relevant systematic reviews, meta-analyses, consensus reports, and eligible clinical studies were additionally screened to identify publications not captured by the electronic search.

Titles and abstracts retrieved through the database searches were screened for relevance to the scope of the review, and potentially relevant publications were subsequently assessed in full text. Publications selected for the clinical biomaterial synthesis were checked against the eligibility framework described below.

The initial screening and selection of publications was performed by J.Ł., and the final selection was reviewed by M.P. for consistency with the predefined scope and eligibility framework. Only publications available in English were included in the narrative synthesis. No restriction to a single publication type was applied because different study designs were required to address the clinical and translational scope of this narrative review; however, primary human clinical evidence was prioritized whenever available. Because the present work was designed as a structured narrative review rather than a systematic review, no formal duplicate independent screening, PRISMA-based study-selection procedure, or systematic risk-of-bias assessment was undertaken.

### 2.2. Eligibility and Evidence Selection

Priority was given to studies directly evaluating regenerative treatment of established peri-implantitis in humans, including randomized controlled trials, prospective and retrospective cohort studies, and clinically relevant case series. Systematic reviews and meta-analyses were used to contextualize the overall evidence and to identify areas of consistency or uncertainty across individual clinical studies.

For the clinical biomaterial synthesis, studies were considered directly relevant when they

1. Included patients with clinically diagnosed peri-implantitis or peri-implant osseous defects associated with peri-implantitis;
2. Evaluated a regenerative surgical intervention involving a grafting material, biologically active agent, platelet concentrate, barrier or space-maintaining device, or a combination of these approaches;
3. Reported at least one clinically relevant outcome, including probing depth, bleeding or suppuration on probing, radiographic bone-level change or defect fill, disease resolution, implant survival, or treatment-related complications; and
4. Provided sufficient information on the study population, intervention, follow-up, and outcome assessment to allow meaningful interpretation.

Studies were excluded from the direct clinical biomaterial synthesis when they evaluated peri-implant mucositis without peri-implant bone loss, primary bone augmentation performed at the time of implant placement in the absence of peri-implantitis, implant surface modifications intended primarily to enhance initial osseointegration, or interventions unrelated to regenerative treatment of an established peri-implant lesion. Publications were also excluded when essential information concerning the study population, intervention, follow-up, or outcome assessment was unavailable.

Preclinical in vitro and animal studies were considered only when they provided relevant mechanistic or translational information that could not be adequately addressed by clinical studies. Evidence derived from periodontal regeneration, endodontic regeneration, or regeneration around natural teeth was not considered direct evidence for peri-implantitis treatment. Where such studies were discussed in relation to emerging cell-based or biologically enhanced strategies, they were explicitly identified as indirect or translational evidence.

### 2.3. Evidence Synthesis and Interpretation

The included literature was synthesized narratively because substantial methodological and clinical heterogeneity precluded meaningful direct comparison across all regenerative strategies. Particular attention was given to study design, sample size, defect morphology, comparator treatment, follow-up duration, clinical inflammatory outcomes, probing depth changes, radiographic defect fill or bone-level changes, implant survival, adverse events, and treatment limitations.

For each biomaterial or regenerative strategy, the evidence was interpreted descriptively according to study design, availability and directness of human peri-implantitis evidence, comparator treatment, sample size, follow-up duration, consistency of reported outcomes, and whether the biomaterial itself represented the randomized treatment variable. The existence of randomized clinical evidence was considered separately from demonstration of superiority for a specific clinical or radiographic outcome. Accordingly, no categorical certainty-of-evidence rating was assigned.

No formal meta-analysis or systematic risk-of-bias assessment was performed. Consequently, the conclusions of this review should be interpreted in light of the inherent limitations of a narrative evidence synthesis, including the potential for study-selection bias, heterogeneity of the included literature, and the absence of formal quantitative certainty assessment.

## 3. Defect Morphology and Clinical Decision-Making

**Figure 1:**
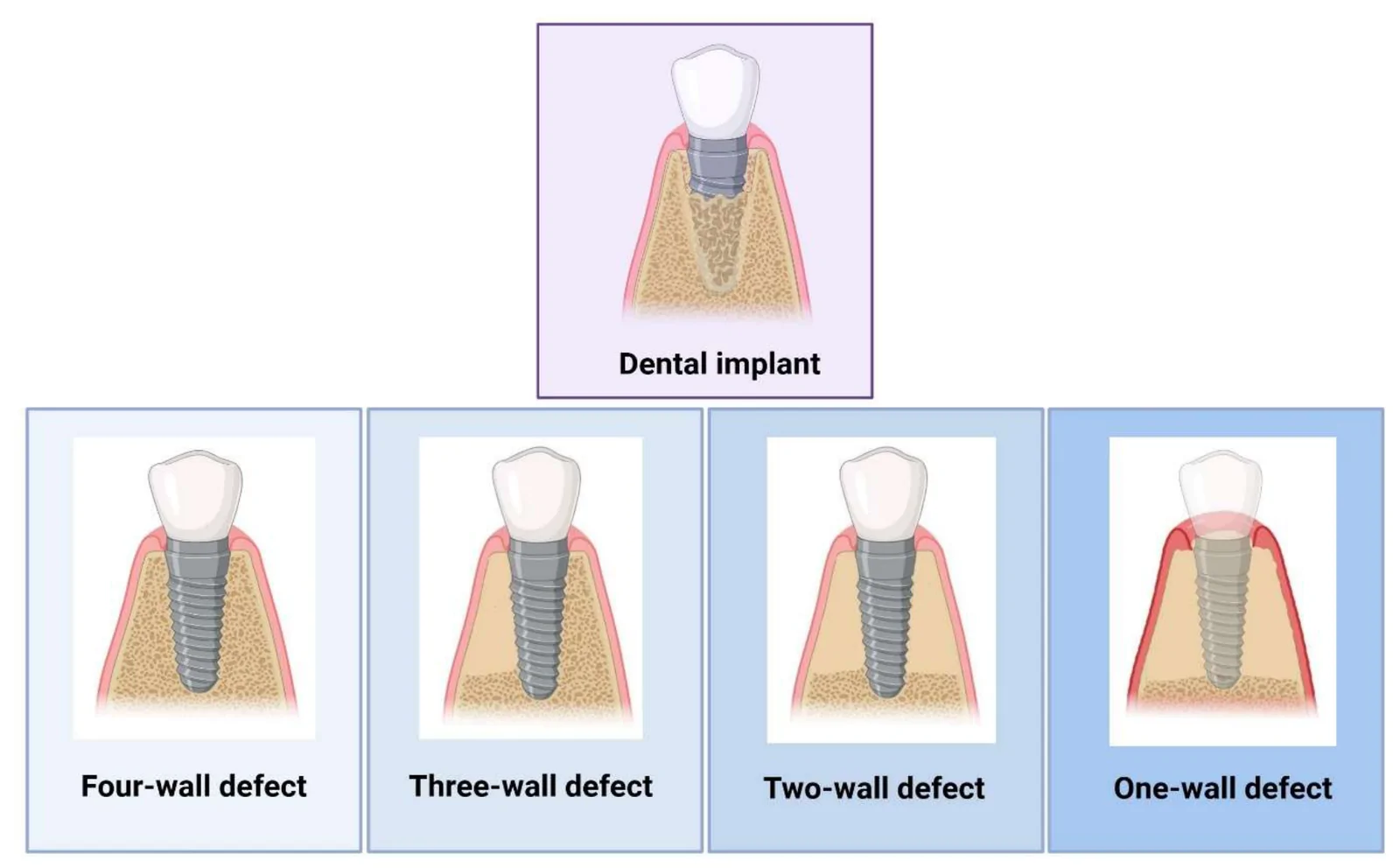
Schematic classification of peri-implant bone defects according to the number of remaining bony walls. Four-wall, three-wall, two-wall, and one-wall defect configurations are illustrated. Defect morphology represents an important factor influencing regenerative potential but should not be considered independently when selecting a treatment strategy (Created in BioRender. Deptuła, M. (2026) https://BioRender.com/l4dnr2v, accessed on 25 July 2026.).

Peri-implant bone defects may present with different configurations depending on the extent and pattern of bone loss. According to the number of remaining bony walls, defects can be described as four-wall, three-wall, two-wall, or one-wall defects (Figure 1) [12].

Additional configurations include dehiscence-type defects, in which the implant surface becomes exposed along a substantial portion of its length, as well as more complex combined defects. The morphology of the defect is clinically relevant because contained defects with a greater number of residual bony walls generally provide more favourable conditions for blood supply, graft containment, and maintenance of the regenerative space [11,12].

However, defect morphology alone should not determine the treatment strategy. Clinical decision-making should incorporate several additional groups of factors [9].

Defect-related factors include the number and height of remaining bony walls, the depth and width of the defect, defect containment, and the possibility of maintaining a stable regenerative space [11].

Implant-related factors include implant mobility, the amount of remaining bone support, implant position, angulation, accessibility of the exposed implant surface for effective decontamination, and the extent of surface exposure [9].

Prosthetic factors include the possibility of restoring the implant in an appropriate prosthetic position, accessibility for long-term oral hygiene, prosthetic contour, and the possibility of modifying or replacing the existing restoration when necessary [8].

Soft-tissue factors include the condition and thickness of the peri-implant mucosa, the presence of inflammation, the amount of keratinized tissue, flap mobility, and the possibility of achieving stable and tension-free wound closure [8,17].

Patient-related factors include oral hygiene, smoking, systemic health, history of periodontal disease, adherence to supportive maintenance, and the patient’s ability to maintain adequate plaque control after treatment [5].

The final treatment decision should therefore result from the combined assessment of defect morphology, implant prognosis, prosthetic feasibility, peri-implant soft-tissue conditions, and patient-related risk factors rather than from the number of remaining bony walls alone [9,11]. Regenerative treatment may be considered when the implant remains stable, the defect provides a reasonable regenerative environment, effective surface decontamination is achievable, and long-term prosthetic and hygienic maintenance is feasible [11]. Conversely, implant removal should be considered when implant mobility, extensive loss of supporting bone, unfavourable implant position, inability to adequately decontaminate the implant surface, poor prosthetic prognosis, or an unfavourable combination of local and patient-related factors makes predictable preservation unlikely [9].

### 3.1. Pre-Surgical Disease Control

The initial phase of peri-implantitis treatment should focus on reducing the inflammatory burden and controlling modifiable local risk factors before surgical intervention is considered. This includes reinforcement of oral hygiene, professional removal of plaque and calculus, correction of prosthetic factors that may impair access for cleaning when feasible, and mechanical debridement of the peri-implant region [8].

Several methods have been proposed for implant surface decontamination, including titanium or non-metallic curettes, ultrasonic instrumentation, air-polishing systems, antiseptic agents, and laser-based approaches. However, the available evidence does not support a universally superior decontamination protocol applicable to all peri-implantitis defect. The selected method should therefore aim to reduce bacterial deposits and inflamed granulation tissue while minimizing additional damage to the implant surface and surrounding peri-implant tissues [18].

The clinical response to initial therapy should be reassessed before proceeding to surgery [8]. Persistent bleeding and/or suppuration on probing, residual deep peri-implant pockets, and progressive or advanced bone loss may indicate the need for surgical access [9]. Surgical treatment allows direct visualization of the affected implant surface and surrounding defect, facilitates more thorough debridement and decontamination, and enables selection of a resective, regenerative, or combined approach according to the local clinical conditions maintenance [9].

Importantly, regenerative surgery should not be regarded as a substitute for adequate disease control. The long-term stability of reconstructive treatment remains dependent on effective plaque control, reduction of inflammation, management of modifiable risk factors, and regular supportive peri-implant maintenance [8].

### 3.2. The Process of Guided Bone Regeneration and Available Biomaterials

Guided bone regeneration (GBR) is based on the creation and maintenance of a protected environment that allows bone-forming cells and vascular structures to repopulate a defect while limiting interference from rapidly proliferating soft tissues [17,19]. In peri-implantitis, this concept is particularly challenging because regeneration is attempted in a site previously affected by inflammation, bacterial contamination, and loss of supporting bone [12].

Successful regenerative therapy therefore depends on several interrelated biological and mechanical condition. These include adequate debridement and decontamination of the affected implant surface, preservation of vascular supply from the surrounding bone, stabilization of the blood clot and grafted compartment, maintenance of sufficient regenerative space, and tension-free wound closure [17]. The morphology of the peri-implant defect strongly influences the ability to fulfil these requirements, as contained defects generally provide better graft stability and vascular support than non-contained defects [11].

Bone substitute materials are used primarily to provide an osteoconductive scaffold and to maintain the volume of the regenerative compartment during healing [20]. Their biological behaviour differs according to composition, porosity, resorption characteristics, mechanical stability, and interaction with the surrounding tissues [20]. Depending on the defect configuration, grafting materials may be used alone or in combination with barrier membranes or rigid space-maintaining devices [19].

Barrier membranes are intended to protect the regenerative compartment from soft-tissue collapse and premature connective-tissue ingrowth while maintaining conditions favourable for bone formation. Both resorbable and non-resorbable membranes are available. Their selection should be based on defect morphology, the need for space maintenance, surgical complexity, and the clinician’s ability to achieve stable soft-tissue coverage. Rigid devices, including titanium-reinforced membranes or meshes, may provide additional space stability in larger or poorly contained defects but may also increase the consequences of postoperative soft-tissue dehiscence or device exposure [19].

Importantly, the presence of a grafting material does not itself constitute evidence of true peri-implant regeneration or re-osseointegration [14]. Clinical and radiographic outcomes such as probing-depth reduction, reduction of bleeding or suppuration, radiographic defect fill, and implant survival provide indirect measures of treatment success, whereas direct bone-to-implant contact can only be demonstrated histologically [14]. Consequently, radiographic defect fill cannot reliably distinguish newly formed vital bone from residual radiopaque graft particles or non-resorbable reconstructive materials and should therefore not be interpreted as direct evidence of re-osseointegration [14].

No single biomaterial or GBR protocol has demonstrated universal superiority in the treatment of peri-implantitis [10]. Clinical outcomes are influenced not only by the selected grafting material but also by defect morphology, implant surface characteristics, decontamination protocol, surgical technique, soft-tissue management, patient-related factors, and supportive maintenance [11]. For this reason, the following sections evaluate individual biomaterials not only according to their theoretical regenerative properties, but also according to the quality and directness of the available clinical evidence in peri-implantitis.

### 3.3. Role of Platelet-Rich Fibrin in Regenerative Therapy

Platelet-rich fibrin (PRF) is an autologous platelet concentrate increasingly used as an adjunct in regenerative procedures in oral and implant surgery [21,22]. It is obtained from the patient’s peripheral blood without the addition of anticoagulants and forms a three-dimensional fibrin network containing platelets, leukocytes, and a range of biologically active mediators [21]. Unlike conventional bone substitute materials, PRF does not primarily function as a space-maintaining scaffold. Its principal role is biological enhancement of the local healing environment [21,22].

The fibrin matrix provides a temporary three-dimensional framework that may facilitate cellular migration and stabilization of the initial blood clot [21]. Platelets and leukocytes incorporated within the fibrin network release growth factors and cytokines, including platelet-derived growth factor (PDGF), transforming growth factor beta (TGF-β), and vascular endothelial growth factor (VEGF), which may contribute to angiogenesis, cellular recruitment, extracellular matrix formation, and soft-tissue healing [21]. These properties provide the biological rationale for combining PRF with conventional grafting materials in regenerative bone procedures [21,22].

Different PRF preparations have been developed, including advanced platelet-rich fibrin (A-PRF) and injectable platelet-rich fibrin (I-PRF) [21]. Injectable preparations may be combined with particulate bone substitutes to form a cohesive graft commonly referred to as “sticky bone”, which can improve handling and initial graft stability [21,22]. Solid PRF membranes may additionally be positioned over grafted sites to support soft-tissue healing and provide a biologically active interface between the grafted compartment and the overlying flap [21,22].

However, PRF membranes should not be regarded as equivalent to conventional barrier membranes used in guided bone regeneration [21,22]. Their relatively rapid degradation and limited mechanical stability restrict their ability to provide prolonged space maintenance or prevent soft-tissue collapse in non-contained defects [21]. Consequently, PRF is better considered a biological adjunct rather than a substitute for structural grafting materials, collagen membranes, or rigid space-maintaining devices when these are required [21,22].

The potential benefit of PRF is particularly relevant in regenerative procedures in which vascularization and early soft-tissue healing may influence treatment outcomes [21]. Nevertheless, the available literature is heterogeneous with regard to PRF preparation protocols, centrifugation conditions, combination with other biomaterials, defect morphology, and clinical outcome measures [22,23]. Direct clinical evidence specifically evaluating PRF in peri-implantitis remains limited to a small number of controlled studies, while a substantial proportion of the broader evidence supporting its regenerative properties originates from other oral regenerative indications [24].

Direct human evidence is provided by Hamzacebi et al. [24], who randomized 19 patients with peri-implant bone loss to surgical treatment with adjunctive PRF or conventional access-flap surgery alone. At 6 months, adjunctive PRF was associated with greater probing-depth reduction and clinical attachment-level gain compared with the control group. However, the small sample size and short follow-up substantially limit the strength and generalizability of these findings [24].

A subsequent systematic review concluded that platelet concentrates may provide favourable clinical effects in peri-implantitis treatment, but emphasized the small number of comparative studies and substantial methodological heterogeneity. Therefore, additional randomized controlled trials are required before a specific independent therapeutic effect can be established [23].

Accordingly, PRF should currently be considered a promising autologous adjunct capable of modifying the biological environment of regenerative sites rather than an independently proven treatment for peri-implantitis [24]. Its potential clinical value is most likely to arise from combination with appropriate defect decontamination, stable grafting materials, space-maintaining strategies, and adequate soft-tissue management [21,23].

## 4. Tissue Engineering and Biologically Enhanced Regeneration

Tissue engineering combines biomaterial scaffolds, biologically active signals, and, in selected experimental approaches, regenerative cells to create an environment capable of supporting tissue repair and reconstruction [15]. In oral regenerative medicine, these principles have been investigated extensively in periodontal therapy, bone augmentation, and implant dentistry [15]. However, the biological objectives of periodontal regeneration around natural teeth and peri-implant regeneration should be clearly distinguished [6,14].

Periodontal regeneration around a natural tooth aims to restore the complex attachment apparatus, including alveolar bone, periodontal ligament, and cementum [15]. Dental implants, in contrast, lack both cementum and a periodontal ligament [6]. Therefore, regenerative treatment of peri-implantitis primarily aims to control inflammation, reconstruct lost peri-implant bone, improve the surrounding soft-tissue environment, and maintain a clinically stable and functional implant [12]. Although restoration of direct bone-to-implant contact may occur experimentally, true re-osseointegration can only be confirmed histologically and should not be assumed from clinical or radiographic bone fill alone [14].

Conventional regenerative biomaterials primarily provide structural and osteoconductive support [20]. Tissue-engineering strategies seek to extend this function by introducing biological activity capable of influencing processes such as angiogenesis, cellular recruitment, inflammatory modulation, extracellular-matrix formation, and osteogenic differentiation [15]. These biological effects may be achieved through bioactive molecules, autologous platelet concentrates, modified scaffolds, or cell-based approaches [15,21].

The clinical relevance of these strategies depends not only on their biological potential but also on the strength and directness of the supporting evidence. Findings obtained from periodontal defects, endodontic regeneration, or preclinical animal and in vitro models may provide valuable mechanistic information, but they should not be considered equivalent to clinical evidence for treatment of peri-implantitis [14]. The following sections therefore distinguish established clinical biomaterials from emerging biologically enhanced and cell-based approaches and evaluate them according to the level of evidence directly applicable to peri-implant tissue regeneration.

### 4.1. Clinical Evidence for Regenerative Biomaterials

A wide range of biomaterials has been investigated for reconstructive treatment of peri-implant bone defects, including enamel matrix derivatives, porous titanium granules, deproteinized bovine bone mineral (DBBM), hydroxyapatite-based materials, and biphasic calcium phosphate (BCP), [25]. These materials differ substantially in their biological function, resorption characteristics, mechanical stability, handling properties, and ability to maintain regenerative space [25].

Importantly, the presence of radiographic bone fill after regenerative surgery should not be interpreted as direct evidence that a particular biomaterial has restored the original bone–implant interface [14]. Most available clinical studies assess surrogate outcomes such as probing-depth reduction, bleeding on probing, radiographic defect fill, marginal bone-level changes, disease resolution, and implant survival [13]. Histological evidence of true re-osseointegration is rarely available in human peri-implantitis studies [14]. Consequently, the clinical effectiveness of regenerative biomaterials should be interpreted primarily according to their ability to contribute to disease control, defect reconstruction, and long-term implant stability rather than to presumed restoration of the original implant–bone interface [25].

Comparison between biomaterials remains difficult because the available studies differ considerably in peri-implantitis case definitions, defect morphology, implant surface characteristics, decontamination protocols, membrane use, surgical technique, outcome definitions, and duration of follow-up [13]. In addition, many regenerative interventions combine several components, such as a bone substitute, barrier membrane, biologically active agent, or local antimicrobial therapy. The observed treatment effect therefore cannot always be attributed to the grafting material alone [25].

For this reason, the following subsections evaluate each biomaterial according to four principal domains: biological function, direct clinical evidence in peri-implantitis, reported clinical and radiographic outcomes, and limitations of the available evidence. Particular emphasis is placed on whether the intervention has demonstrated benefit compared with open-flap debridement, another regenerative material, or an alternative reconstructive strategy rather than simply whether improvement was observed after treatment.

The available evidence does not currently support a single universally superior regenerative material for all peri-implantitis defects [10,11,25]. Clinical outcomes appear to depend on the interaction between defect configuration, implant stability and position, effectiveness of implant-surface decontamination, soft-tissue management, graft stability, and patient-related factors in addition to the selected biomaterial [11,25]. Accordingly, biomaterial selection should be considered one component of a broader regenerative strategy rather than an independent determinant of treatment success [11,25].

Table 1 provides a comparative summary of the principal clinical studies evaluating regenerative biomaterials in peri-implantitis, including study design, sample size, comparator, follow-up period, reported clinical and radiographic outcomes, and major methodological limitations. Table 2 summarizes the principal biological, structural, and clinical characteristics of the evaluated regenerative materials, including their biological role, resorption or persistence profile, space-maintaining capacity, direct clinical evidence in peri-implantitis, major advantages and limitations, and relative resource burden.

#### 4.1.1. Emdogain^®^

Enamel matrix derivative (EMD; Emdogain^®^ Institut Straumann AG, Basel, Switzerland) is a biologically active preparation originally developed to support periodontal regeneration [45]. Its principal protein components are amelogenin-related proteins, although EMD has also been associated with modulation of growth-factor signalling and cellular responses involved in wound healing and tissue regeneration [46,47]. Experimental and periodontal studies suggest that EMD may influence fibroblast activity, angiogenesis, extracellular matrix formation, and the local inflammatory environment. However, evidence derived from periodontal regeneration around natural teeth should not be interpreted as direct evidence of regeneration around dental implants, where neither cementum nor periodontal ligament is present [6].

Direct clinical evidence for EMD in the treatment of peri-implantitis is considerably more limited than the periodontal literature. In a randomized clinical trial, Isehed et al. evaluated surgical treatment of peri-implantitis with or without adjunctive EMD and reported favourable long-term implant survival in the EMD-treated group during 3- and 5-year follow-up [26]. The study provides clinically relevant long-term information; however, the relatively small sample size and the use of EMD as an adjunct to surgical treatment limit the ability to attribute the observed effect exclusively to the material itself.

The original 12-month report from the same randomized cohort included 26 patients undergoing surgical treatment of peri-implantitis with or without adjunctive EMD [33]. Multivariate analysis demonstrated an association between EMD treatment and increased marginal bone level, whereas between-group differences in probing depth, bleeding, suppuration, and other clinical parameters were not statistically significant in univariate analyses. These findings suggested a potential radiographic benefit of adjunctive EMD but did not establish a consistent clinical advantage across the evaluated peri-implantitis outcomes.

Additional supportive human evidence was provided by Wilson et al. in a consecutive case series of 25 patients involving 34 implants treated surgically with adjunctive EMD [48]. After mean follow-up of approximately 3 years, 94% of the treated implants remained in function, accompanied by significant reductions in probing depth and bleeding on probing. However, the uncontrolled study design, absence of a comparator group, and simultaneous surgical intervention substantially limit causal interpretation. The study therefore supports the clinical feasibility of EMD-containing surgical protocols but does not demonstrate superiority over alternative regenerative approaches.

More recent randomized evidence has questioned whether EMD provides an additional benefit when incorporated into an established reconstructive protocol. Regidor et al. randomized 40 patients involving 44 implants to reconstructive surgery using a bone graft and resorbable membrane with or without adjunctive EMD [35]. At 12 months, mean probing-depth reduction was 4.3 ± 2.4 mm in the EMD group and 4.0 ± 1.7 mm in the control group (*p* = 0.105). No significant between-group differences were observed in clinical, radiographic, composite, or patient-reported outcomes. These findings indicate that the addition of EMD to graft- and membrane-based reconstructive therapy did not provide a detectable incremental clinical benefit in this trial.

Biological observations may provide additional mechanistic support for EMD as an adjunctive agent. A systematic review evaluating biomarker expression in peri-implantitis lesions reported changes in inflammatory mediators following treatment involving EMD [49]. However, biomarker modulation should not be interpreted as equivalent to clinical disease resolution, peri-implant bone regeneration, or restoration of the bone–implant interface.

At the evidence-synthesis level, a systematic review of human clinical studies concluded that the literature evaluating EMD in peri-implantitis remains limited and heterogeneous [50]. Although favourable outcomes have been reported in both surgical and non-surgical applications, the available evidence was considered modest for surgical treatment, and further randomized trials using standardized protocols were recommended.

Overall, EMD has a biologically plausible role as an adjunct in regenerative peri-implantitis surgery, but its independent therapeutic contribution remains uncertain. Earlier randomized and observational studies suggested potential radiographic or long-term survival benefits, whereas a more recent randomized controlled trial failed to demonstrate a significant incremental benefit when EMD was added to a bone graft and resorbable membrane [26,33,35]. Current evidence therefore does not establish EMD as an independently effective regenerative treatment or demonstrate consistent superiority over reconstructive surgery without EMD. Further adequately powered randomized trials using standardized peri-implantitis definitions, defect classifications, surgical protocols, and clinically meaningful outcome measures are required to determine whether specific patient or defect subgroups benefit from adjunctive EMD.

Evidence interpretation: Direct human clinical evidence includes randomized controlled trials, observational studies, and long-term follow-up data. However, findings are inconsistent, and EMD is generally evaluated as an adjunct within multimodal surgical protocols rather than as an isolated intervention. Current evidence supports biological plausibility and clinical feasibility but does not establish a consistent incremental benefit of EMD in reconstructive peri-implantitis therapy [26,33,35,50].

#### 4.1.2. Porous Titanium Granules

Porous titanium granules (PTGs) are non-resorbable alloplastic materials proposed for reconstructive treatment of peri-implant osseous defects because of their biocompatibility, high porosity, and ability to provide mechanical stability and maintain defect volume. Their principal regenerative role is predominantly osteoconductive and structural rather than osteogenic, providing a stable porous framework that may support ingrowth of mineralized tissue while remaining permanently within the reconstructed site [25,40]. Recent reviews of reconstructive peri-implantitis therapy similarly characterize PTGs as highly porous, osteoconductive, and non-resorbable materials.

Direct randomized clinical evidence was first provided by Wohlfahrt et al. [40], who evaluated PTGs in the surgical treatment of peri-implant osseous defects. Reconstruction with PTGs resulted in significantly greater radiographic defect-height reduction and percentage defect fill compared with the control procedure. Both groups demonstrated improvements in probing-pocket depth, but no significant between-group differences were observed in the clinical periodontal parameters. Importantly, the authors emphasized that the greater radiographic defect fill should not be interpreted as proof of re-osseointegration or osseointegration of individual titanium granules [40].

Supportive observational evidence was reported by Mijiritsky et al. [41] in a retrospective cohort comprising 18 implants in 16 patients. Treatment consisted of open-flap debridement, tetracycline-based surface decontamination, reconstruction with PTGs, and apically positioned flaps. After a mean follow-up of 7.5 months, 16 of 18 implants fulfilled the authors’ treatment-success criteria, corresponding to an overall success rate of 88%. Mean radiographic bone loss decreased from 4.4 ± 2.1 mm before treatment to 2.3 ± 2.1 mm at follow-up. However, the retrospective design, small sample, short and variable follow-up, absence of a comparator group, and use of a composite definition of success substantially limit the strength of these findings [41].

More robust comparative evidence was subsequently provided by Jepsen et al. [27] in a multinational multicentre randomized controlled trial involving 63 patients with circumferential peri-implant intraosseous defects. At 12 months, open-flap debridement combined with PTGs resulted in mean radiographic defect fill of 3.6 mm both mesially and distally, compared with 1.1 and 1.0 mm, respectively, following open-flap debridement alone (*p* < 0.0001). However, mean probing-depth reduction was similar between groups, and complete disease resolution was achieved in 30% of PTG-treated patients compared with 23% of controls, without a significant between-group difference. These findings demonstrate a clear radiographic advantage of PTGs but a substantially less pronounced incremental effect on clinical disease resolution [27].

Guler et al. [28] further compared a PTG-based reconstructive protocol with a xenogeneic graft-based approach in 22 patients with peri-implantitis. Clinical attachment levels improved in both groups, from 5.29 ± 1.06 to 3.59 ± 0.88 mm in the PTG group and from 4.77 ± 1.05 to 3.30 ± 0.58 mm in the xenograft group. Radiographic bone filling was significantly greater in the PTG group. Nevertheless, the two treatment protocols differed in several components: the PTG group received rotary titanium-brush debridement, PTGs, and a PRF membrane, whereas the comparator group received a xenograft, collagen membrane, and PRF membrane. Consequently, the observed differences cannot be attributed exclusively to the grafting material itself [28].

A recent review of biomaterials for reconstructive peri-implantitis therapy reached a similar overall interpretation: randomized studies of titanium granules have demonstrated greater radiographic defect fill or improved radiographic bone levels compared with non-reconstructive treatment, whereas changes in bleeding on probing and probing depth have generally been comparable between treatment groups [25].

An important limitation in interpreting PTG studies is the permanent presence of the non-resorbable titanium granules within the reconstructed compartment. Consequently, increased radiopacity or apparent defect fill cannot be assumed to represent newly formed vital bone or restoration of the original bone–implant interface. Clinical interpretation should therefore emphasize inflammatory disease control, probing parameters, implant retention, and long-term stability in addition to radiographic reconstruction.

Overall, porous titanium granules represent a mechanically stable and osteoconductive reconstructive option for selected peri-implant osseous defects. Direct randomized and prospective clinical evidence consistently indicates that PTGs can increase radiographic defect fill compared with open-flap debridement alone. However, a corresponding superiority in clinical disease resolution has not been demonstrated consistently, and comparative evidence against alternative grafting materials remains limited by small samples and differences between treatment protocols. Their principal clinical advantage therefore appears to be maintenance of defect volume and radiographic reconstruction rather than demonstrated biological superiority over other regenerative materials.

Evidence interpretation: Direct human evidence includes randomized controlled trials and prospective comparative studies, with consistent support for improved radiographic defect reconstruction. However, clinical inflammatory outcomes and complete disease resolution have not shown similarly consistent superiority, and the non-resorbable nature of PTGs complicates interpretation of radiographic defect fill [25,27,28,40].

#### 4.1.3. DBBM

Deproteinized bovine bone mineral (DBBM) is a xenogeneic bone substitute widely used in oral regenerative procedures because of its biocompatibility, porous mineral structure, and slow resorption profile. Its principal biological role is osteoconductive rather than osteogenic, providing a mineral scaffold that supports vascular and cellular ingrowth from the surrounding recipient bone while contributing to maintenance of the regenerative space during healing [20,25].

The slow resorption and long-term persistence of DBBM may be advantageous where maintenance of graft volume is required. However, residual graft particles may remain within reconstructed sites for prolonged periods. Consequently, radiographic defect fill after DBBM-based reconstruction should not be interpreted as complete replacement by newly formed vital bone or as direct evidence of re-osseointegration [14,20].

Long-term direct clinical evidence is provided by the prospective cohort reported by Roccuzzo et al. [42]. In the original population of 26 patients with peri-implantitis intrabony defects treated using DBBM containing 10% collagen (DBBMC), substantial improvements in probing depth and inflammatory parameters were maintained in many implants at 7 years [42]. However, treatment outcomes were strongly influenced by implant-surface characteristics. Treatment success, defined as probing depth ≤5 mm, absence of bleeding or suppuration, and absence of further bone loss, was achieved in 58.3% of SLA implants compared with 14.3% of TPS implants. Several patients required additional antibiotic or surgical treatment during supportive care, demonstrating that reconstructive surgery did not reliably eliminate the risk of disease recurrence.

Ten-year follow-up of the same cohort further demonstrated the challenges of long-term disease control [30]. Fourteen of the original 26 patients attended the 10-year examination. Overall implant survival was 67%, with survival rates of 80% for SLA and 55% for TPS implants. Mean probing depth decreased from 6.6 ± 1.3 to 3.2 ± 0.7 mm for SLA implants and from 7.2 ± 1.5 to 3.4 ± 0.6 mm for TPS implants, whereas treatment success was achieved in 42% and 29% of SLA and TPS implants, respectively. These findings provide valuable long-term evidence but also demonstrate that implant survival should not be equated with complete disease resolution. Substantial attrition, absence of an untreated comparator, additional retreatment during supportive care, and the influence of implant-surface characteristics limit causal interpretation.

Mercado et al. [31] evaluated a multimodal regenerative protocol combining DBBMC, EMD, and doxycycline in a prospective cohort of 30 patients with peri-implantitis [31]. At 36 months, mean probing depth decreased from 8.9 ± 1.9 to 3.50 ± 0.50 mm, while mean radiographic bone loss decreased from 6.92 ± 1.26 to 2.60 ± 0.73 mm. The predefined successful treatment outcome was achieved in 56.6% of treated implants. However, the absence of a control group and simultaneous use of DBBMC, EMD, doxycycline, surface decontamination, and, where required, connective-tissue grafting prevent attribution of the observed effect to DBBM alone. The study therefore provides direct clinical evidence for the combined regenerative protocol rather than for the independent efficacy of DBBM [31].

Higher-level comparative evidence was provided by Renvert et al. [29] in a multicentre randomized controlled trial comparing surgical debridement alone with reconstructive treatment using DBBM and a native bilayer collagen membrane [29]. At 12 months, radiographic defect fill at the deepest site was significantly greater following DBBM-based reconstruction than after debridement alone (2.7 ± 1.3 vs. 1.4 ± 1.2 mm; *p* < 0.0001). However, mean probing-depth reduction was not significantly different between groups, and no significant advantages were observed for bleeding on probing, suppuration, mucosal recession, or patient-reported outcomes. Composite treatment success was achieved in 32% of the reconstructive group and 21% of controls. These results therefore demonstrate a clear radiographic benefit without a corresponding consistent superiority across clinical disease-resolution endpoints [29].

The 3-year follow-up of the same randomized cohort showed that the improvements achieved during the first year were generally maintained during supportive peri-implant therapy [34]. At 36 months, mean radiographic defect fill was 2.13 ± 1.26 mm following DBBM and membrane-based reconstruction compared with 1.64 ± 1.54 mm following open-flap debridement alone; the difference was no longer statistically significant (*p* = 0.18). No significant between-group differences were observed in probing depth, bleeding on probing, suppuration, mucosal recession, or patient-reported outcomes. Composite treatment success was achieved in 46.2% of the reconstructive group and 20.0% of controls, although this difference narrowly failed to reach conventional statistical significance (*p* = 0.053). These findings further emphasize the distinction between radiographic defect reconstruction and complete clinical disease resolution [34].

Additional case-based evidence has described modified DBBM protocols. Bhatavadekar and Gharpure reported the use of DBBM with collagen in combination with locally selected antibiotics and a resorbable collagen membrane for peri-implantitis defects [51]. Although favourable clinical and radiographic outcomes were reported, the very small sample, absence of a comparator group, and use of several therapeutic components substantially limit conclusions regarding material-specific effectiveness.

Overall, DBBM is one of the more extensively investigated osteoconductive grafting materials in reconstructive peri-implantitis therapy. Prospective studies, randomized controlled trials, and long-term follow-up data support its ability to contribute to radiographic defect reconstruction and improvement of selected peri-implant clinical parameters. Nevertheless, the available evidence consistently demonstrates an important discrepancy between radiographic defect fill and complete clinical disease resolution. Moreover, DBBM is frequently used together with collagen membranes, biologically active agents, antimicrobial therapy, or other surgical interventions, making its independent contribution difficult to isolate. Current evidence therefore supports DBBM as a clinically established reconstructive scaffold but does not demonstrate universal superiority over alternative materials or open-flap surgery across all clinically relevant outcomes [29,30,31,34,42].

Evidence interpretation: Direct human evidence includes prospective cohorts, randomized controlled trials, and follow-up extending to 7–10 years. The evidence consistently supports radiographic reconstructive effects, but disease resolution remains variable, material-specific effects are often difficult to isolate, and long-term outcomes are influenced by implant surface, supportive maintenance, retreatment, and other clinical factors [29,30,34,42].

#### 4.1.4. Hydroxyapatite

Hydroxyapatite (HA) is a calcium phosphate ceramic with a chemical composition similar to the mineral phase of native bone. Owing to its biocompatibility and osteoconductive properties, HA has been widely used in oral bone-regeneration procedures in the form of granules, porous scaffolds, composites, and nanocrystalline formulations. Its principal regenerative function is to provide a mineral scaffold supporting cellular attachment and ingrowth of newly formed bone rather than to provide intrinsic osteogenic activity [25,52].

The biological behaviour of HA depends substantially on its physicochemical characteristics, including crystallinity, porosity, particle size, origin, and incorporation into composite materials. Highly crystalline HA may persist for prolonged periods, contributing to maintenance of graft volume but also complicating radiographic interpretation because residual particles may remain visible within the reconstructed defect. Consequently, radiographic mineral density or defect fill should not automatically be interpreted as complete replacement of the biomaterial by newly formed vital bone or as evidence of re-osseointegration [14,52].

Direct clinical evidence for HA-based reconstruction in peri-implantitis includes several controlled human studies. Schwarz et al. [36] evaluated nanocrystalline hydroxyapatite (NHA) in a randomized clinical cohort of patients with peri-implantitis intrabony defects and compared it with a natural bone mineral used in combination with a collagen membrane. At 48 months, the NHA group demonstrated a mean probing-depth reduction of 1.1 ± 0.3 mm and clinical attachment-level gain of 0.6 ± 0.5 mm, compared with 2.5 ± 0.9 mm and 2.0 ± 1.0 mm, respectively, in the natural bone mineral plus collagen membrane group. Radiographic defect fill was observed at five NHA-treated sites and eight comparator sites. The authors considered the long-term outcome of NHA without a barrier membrane unsatisfactory. However, interpretation is limited because the groups differed not only in the grafting material but also in membrane use, preventing isolation of a material-specific effect [36].

Stronger evidence for HA-based defect reconstruction was subsequently provided by Renvert et al. [37] in a randomized controlled trial involving 41 patients with three- or four-wall peri-implant bone defects. Surgical debridement alone was compared with debridement followed by reconstruction using bovine-derived hydroxyapatite particles. Radiographic defect fill was significant in the HA-treated group, and the predefined composite treatment outcome was achieved in 42.9% of patients receiving the bone substitute compared with 5.0% of controls (*p* < 0.01). However, absence of bleeding on probing was observed in 47.6% and 35.0% of patients, respectively, without a significant between-group difference. These findings suggest that HA-based reconstruction may improve radiographic and composite treatment outcomes in contained peri-implant defects, although superiority was not consistently demonstrated across individual inflammatory clinical parameters [37].

Polymeri et al. [38] further compared two bovine-derived xenograft materials in a randomized clinical pilot study involving 24 patients with peri-implantitis. Bovine-derived hydroxyapatite (EndoBon^®^, Biomet France SARL, Valence, France) was compared with deproteinized bovine bone mineral (Bio-Oss^®^, Geistlich Pharma AG, Wolhusen, Switzerland) Both groups demonstrated significant clinical and radiographic improvements at 12 months, including mean intrabony defect-depth reductions of approximately 2.5–3.0 mm. No statistically significant between-group differences were observed for the evaluated clinical or radiographic parameters. Using a stringent composite definition of treatment success, successful outcomes were observed in 0 of 13 patients treated with bovine hydroxyapatite and 2 of 11 patients treated with DBBM (*p* = 0.199). The small sample and pilot design limit definitive conclusions, but the findings indicate comparable reconstructive performance rather than superiority of HA over DBBM [38].

Soliman evaluated a nanocrystalline HA-based bone substitute containing nanoporous silica (NanoBone^®^, ARTOSS GmbH, Rostock, Germany) in a randomized clinical trial involving 30 patients with peri-implantitis. Both groups received the HA-based scaffold, while the experimental group additionally received locally applied simvastatin [32]. Clinical and radiographic parameters improved in both groups after six months, with greater radiographic improvement observed following the addition of simvastatin. Because HA was used in both treatment arms, however, the study primarily evaluated the adjunctive effect of simvastatin rather than the independent efficacy of HA-based reconstruction compared with non-grafted surgery.

Additional supportive evidence is provided by Pilenza et al., who retrospectively evaluated 11 patients involving 20 implants treated with a multimodal regenerative protocol combining HA, EMD, and a collagen membrane [43]. At one year, all implants remained in function, mean probing depth decreased from 4.9 to 2.7 mm, bleeding on probing decreased from 90% to 20%, and suppuration decreased from 65% to 0%. Based on the authors’ predefined criteria, 15 of 20 implants (75%) were considered successfully treated. Nevertheless, the absence of a control group and simultaneous use of HA, EMD, collagen membrane, surface decontamination, and systemic antibiotic therapy prevent determination of the specific contribution of HA to these outcomes.

Studies evaluating HA coatings applied to dental implants primarily to enhance osseointegration after implant placement should be distinguished from studies evaluating HA as a reconstructive biomaterial for established peri-implantitis [52]. Such implant-surface studies were therefore not considered direct therapeutic evidence in the present clinical synthesis.

Overall, HA-based materials have demonstrated the capacity to support peri-implant defect reconstruction, but their clinical performance varies substantially according to material formulation and accompanying regenerative protocol. Randomized studies provide evidence of radiographic improvement compared with open-flap debridement and broadly comparable outcomes to other xenogeneic grafting materials. However, HA has not demonstrated consistent superiority across probing, inflammatory, radiographic, and composite disease-resolution outcomes. Moreover, differences between nanocrystalline synthetic HA, bovine-derived HA, composite HA-based materials, membrane use, and adjunctive biologics substantially limit direct comparison between studies [25,36,38,53]. The most recent synthesis likewise concludes that the ideal reconstructive biomaterial for peri-implantitis remains uncertain.

Evidence interpretation: Direct human evidence includes randomized controlled trials, comparative clinical studies, and follow-up extending to four years. However, study populations are generally small, HA formulations differ considerably, several interventions incorporate additional membranes or biologically active agents, and consistent superiority over open-flap debridement or established alternative graft materials has not been demonstrated [25,36,38,53].

#### 4.1.5. Biphasic Calcium Phosphate

Biphasic calcium phosphate (BCP) is a synthetic ceramic biomaterial composed of hydroxyapatite (HA) and β-tricalcium phosphate (β-TCP) in variable proportions. The combination is intended to balance the relatively slow resorption and structural stability of HA with the greater solubility and faster degradation of β-TCP. BCP therefore acts primarily as an osteoconductive scaffold, while its resorption profile can be modified by altering the HA/β-TCP ratio [25,54,55]. Contemporary reviews similarly describe HA/β-TCP composites as alloplastic osteoconductive materials with a biphasic resorption profile intended to approximate physiological bone remodelling.

The biological rationale for BCP is based on maintaining a three-dimensional porous scaffold that supports vascular and cellular ingrowth while permitting progressive replacement of the more soluble component by newly formed mineralized tissue. Higher HA content generally favours longer-term structural persistence, whereas a greater β-TCP fraction increases resorption. However, these properties should not be interpreted as intrinsic osteogenic activity, and the clinical behaviour of BCP depends additionally on defect morphology, vascular supply, graft stability, surface decontamination, and the surrounding biological environment [25,54,55].

Direct clinical evidence for BCP-containing reconstructive protocols in established peri-implantitis is provided by de Tapia et al. [39]. In a randomized double-blind clinical trial involving 30 patients, peri-implantitis defects in both treatment groups were reconstructed using an alloplastic HA/β-TCP graft and covered with a collagen membrane. The randomized intervention concerned implant-surface decontamination: the test group received additional treatment with a titanium brush, whereas the control group underwent mechanical and chemical decontamination without the brush [39]. At 12 months, probing-depth reduction at the deepest site was 4.87 ± 1.55 mm in the titanium-brush group compared with 2.85 ± 1.91 mm in the control group (*p* = 0.009), with residual probing depths of 3.60 ± 0.91 and 4.92 ± 1.26 mm, respectively (*p* = 0.007). These findings demonstrate that favourable clinical outcomes can be achieved with an HA/β-TCP-based reconstructive protocol, but because BCP and the collagen membrane were used in both groups, the trial does not establish the independent therapeutic effect of BCP itself.

Five-year observational follow-up of the same randomized cohort provides valuable longer-term information [44]. Seventeen patients remained available for evaluation. Probing-depth reductions of 2.86 ± 2.27 mm in the original control group and 4.10 ± 1.79 mm in the titanium-brush group were maintained at 5 years, while radiographic bone fill corresponded to 32.77% and 54.91% of the original defect, respectively. Between-group differences were no longer statistically significant, and radiographic bone loss of less than 0.5 mm occurred during the extended follow-up. The substantial attrition and the fact that both groups received the same BCP-based reconstruction again prevent conclusions regarding material-specific superiority, but the study provides evidence that clinical improvements following an HA/β-TCP-containing reconstructive protocol may be maintained over several years.

Additional evidence concerning BCP comes from peri-implant bone-regeneration studies that are clinically relevant but not directly representative of established peri-implantitis. Lee et al. compared a synthetic BCP containing 60% HA and 40% β-TCP with DBBM in a prospective multicentre randomized controlled trial involving 53 peri-implant dehiscence defects [56]. After an 18-week healing period, complete defect resolution was observed in 59.3% of BCP-treated sites and 42.3% of DBBM-treated sites, while mean defect resolution was 71.3% and 57.9%, respectively. Changes in horizontal bone thickness were −0.50 ± 0.66 mm with BCP and −0.66 ± 0.83 mm with DBBM (*p* = 0.133), and no significant differences were observed in postoperative discomfort or early wound healing. These results support the reconstructive performance of BCP as an alternative to DBBM in GBR but should be considered indirect evidence for peri-implantitis because the defects were associated with implant placement rather than inflammatory peri-implant disease.

Garunov et al. evaluated HA and tricalcium phosphate, including a formulation modified with hyaluronic acid, in 128 patients diagnosed with peri-implant mucositis or peri-implantitis [57]. At 12 months, mean peri-implant bone resorption was 0.682 ± 0.006 mm in the hyaluronic-acid-modified calcium phosphate group, compared with 1.025 ± 0.034 mm with unmodified HA/TCP and 1.626 ± 0.022 mm in the control group (*p* < 0.001). Implant-stability measurements were also higher in the modified-material group. Although these findings suggest that biological modification of calcium-phosphate scaffolds may influence peri-implant healing, interpretation is limited by the inclusion of both peri-implant mucositis and peri-implantitis, the use of a modified material, and the absence of sufficiently detailed disease-specific outcomes separating the two diagnoses. The study therefore cannot establish the efficacy of conventional BCP specifically for established peri-implantitis.

An additional challenge in interpreting the BCP literature is the substantial heterogeneity of the materials themselves. Differences in HA/β-TCP ratio, porosity, particle size, crystallinity, scaffold architecture, and incorporation of collagen or biologically active agents can substantially influence degradation and biological behaviour. Consequently, different products described collectively as “BCP” should not automatically be considered clinically equivalent [25,54].

Overall, BCP represents a well-established synthetic osteoconductive scaffold with adjustable degradation characteristics and the advantage of avoiding xenogeneic tissue sources. Direct human peri-implantitis studies demonstrate that BCP-containing reconstructive protocols can be associated with substantial probing-depth reduction and radiographic defect reconstruction, including maintenance of improvements during long-term follow-up [39,44]. However, the available studies generally evaluate BCP as one component of multimodal treatment protocols, and the material itself has rarely been isolated as the randomized variable. Comparative evidence from non-inflammatory peri-implant defects suggests performance broadly comparable with DBBM, but this cannot be directly extrapolated to established peri-implantitis [56]. Therefore, current evidence supports BCP as a clinically plausible synthetic grafting option but does not demonstrate superiority over xenogeneic grafts or establish a specific independent effect on peri-implantitis disease resolution.

Evidence interpretation: Human clinical evidence is available, including randomized and long-term studies involving BCP-containing reconstructive protocols. Nevertheless, material-specific evidence remains limited because BCP has usually been combined with membranes and standardized surgical interventions, while the randomized comparison has frequently concerned another component of treatment rather than BCP itself. Additional trials directly comparing BCP with open-flap debridement and established alternative grafting materials in well-defined peri-implantitis defects are required [25,39,44,57].

### 4.2. Stem Cells in Peri-Implantitis and Periodontal Treatment

Current research is ongoing on a cellular approach utilizing MSCs, which are able to differentiate into the cells from the mesodermal linages like osteocytes, chondrocytes and adipocytes. From a clinical perspective, the use of stem cells represents a promising advancement in regenerative procedures. However, limited availability and the need for specialized laboratories restrict their utilization. Preclinical studies are currently being conducted in the treatment of bone defects using MSCs [58,59]. These cells could be used to treat or prevent peri-implatitis, but direct clinical evidence is lacking at this stage. Most studies are in the preclinical level and involve in vitro analyses and animal models. However, the available clinical and preclinical data regarding the use of various types of MSCs in periodontal treatment may indirectly indicate their potential use in peri-implant surgery as well.

#### 4.2.1. Stem Cells in Peri-Implantitis

**Figure 2:**
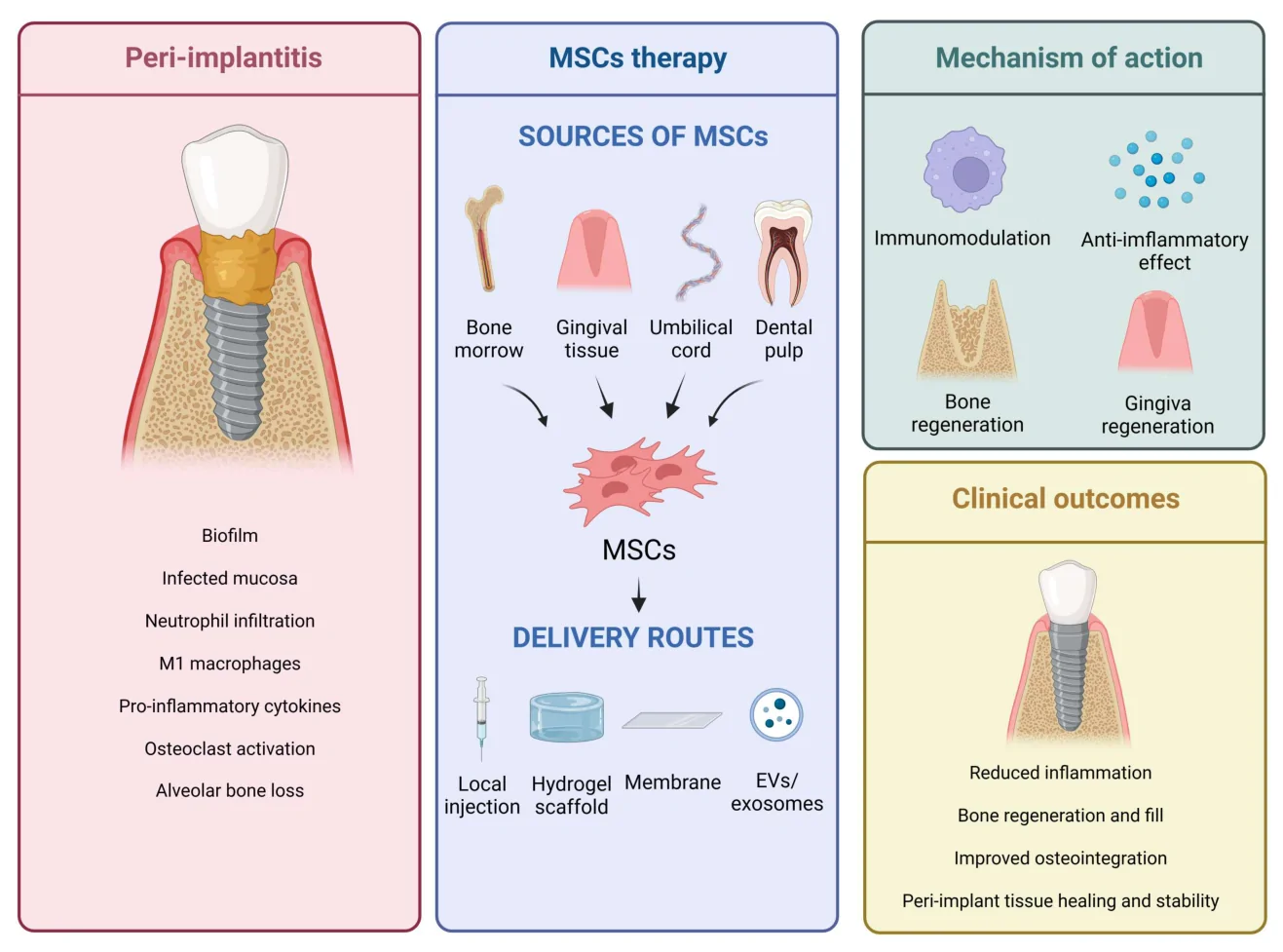
Proposed biological mechanisms and regenerative pathways associated with MSCs-based therapies in peri-implantitis. The illustrated effects are primarily derived from preclinical and translational studies and should not be interpreted as established clinical outcomes in peri-implantitis patients. Created in BioRender. Deptuła, M. (2026) https://BioRender.com/7wv9qia, accessed on 25 July 2026.

At this stage of research, there is not much evidence to confirm the effectiveness of stem cells in the treatment or prevention of peri-implantitis. However, some preclinical data is available. For example, Hendrijantini et al. [60] evaluated the effect of human umbilical cord-derived mesenchymal stromal cells (hUC-MSCs) on the osteointegration of the implant in peri-implantitis rats. In the study the cells were injected 1 mm from the mesial and distal of the implant site. The results show that after 4 weeks, the expression of IL-10, a potent anti- inflammatory cytokine, was significantly higher than in the control group. Significant increase in the expression of TGF-beta, BMP-2, osterix (Osx), and osteoprotegerin (OPG) were also observed in rats threated with cells. The study showed that hUC-MSCs are able to maintain inflammation at the low levels, enhance osteogenesis and implant osteointegration. What is more, available data show that MSCs may potentially improve gingiva regeneration, what is also important in peri- implantitis (Figure 2) [61].

Attempts are also being made to administer stem cells after their prior modification or together with other pro-regenerative substances. For example, a Brazilian mini pig model was used to evaluate the effect of combination of autologous BM-MSCs and PRP on the osteointegration of dental implant. Undifferentiated BM-MSCs with PRP were placed in post-extraction sockets before implant placement. The results showed, non-significant lower implant lost rate, but no positive effect on bone regeneration and osteointegration was observed in treated group compared to the control [62]. In another study, hypoxia pre-conditioned hUC-MSCs were injected 1 mm from the proximal and distal side of the implant in rats with peri-implantitis. A decrease in the expression of TNF-alpha and NFATc1 were observed. TNF-alpha is a pro-inflammatory cytokine correlated with peri-implantitis and NFATc1 is a crucial regulator of osteoclast differentiation. These findings suggests that hypoxia pre-conditioned hUC-MSCs can reduce inflammation, osteoclastic activity and therefore prevent bone resorption in peri-implantitis. Additionally, higher COL1α1 expression was observed in hypoxia and normoxia hUC-MSCs groups, but the effect was visible 14 days earlier in hypoxia pre-conditioned cells. Moreover, an increase in the expression of osteocalcin was observed, and similarly to COL1α1, it was visible earlier in hypoxia group. These results indicate that hypoxia pre-conditioned hUC-MSCs are able to promote bone regeneration on rat model of peri-implantitis [63].

Hydrogels are also tested as carriers for the cells, but their application can be limited due to specific environment in the oral cavity, where they may be exposed to blood and saliva. This means that it is also necessary to develop new hydrogels that can be used as cell carriers in peri-implantitis. Hasani-Sadrabadi et al. [64] engineered a new alginate-based adhesive, photocrosslinkable, and osteoconductive hydrogel, which was used for the encapsulation of gingival mesenchymal stem cells (G-MSCs). This hydrogel containing autologous G-MSCs aggregates/Hydroxyapatite microparticles (Hap MPs) was injected around the periphery of implants in a rat peri-implantitis model, polymerized with a visible light–curing unit, and the flap was covered over the implant. Hap MPs were incorporated to induce osteogenesis. The application of the hydrogels resulted in complete filling of the bony defects and bone regeneration around dental implants. Additionally, sustained reduction in local inflammation was observed. Additionally, Li et al. [65] demonstrated that G-MSCs delivered in porous GelMA/SilMA hydrogels enhance the epithelial seal around implants and promote M2 macrophage polarization. These results suggest that MSCs may limit the development of peri-implantitis not only through immunomodulation but also by improving the integrity of the mucosal barrier around implants [65].

In recent years, regenerative medicine has seen a surge in interest in the use of extracellular vesicles (EVs) instead of cells themselves. This allows for the avoidance of some of the challenges associated with cell transplantation, such as survival and regulatory issues. At the same time, EVs possess unique biological properties, including the ability to influence proliferation, migration, and regulate the immune response. They are also more stable and less immunogenic than cells themselves [66]. Recently, exosomes from umbilical cord blood demonstrated therapeutic efficacy in rat peri-implantitis model. Their application reduced inflammation and enhanced bone regeneration. These findings suggest that EV-based therapies may represent a promising alternative or adjunct to MSC transplantation in peri-implantitis [67].

#### 4.2.2. MSCs in Periodontal Treatment

One of the sources of MSCs used in periodontal treatment are dental MSCs. They can be divided into dental pulp mesenchymal stromal cells (DP-MSCs), dental follicle mesenchymal stromal cells (DF-MSCs), stem cells from exfoliated deciduous teeth (SHED), gingival mesenchymal stromal cells (G-MSCs), and stem cells from apical papilla (SCAPs) [68]. Most commonly used types of dental MSCs in periodontitis are described below.

##### DP-MSCs

DP-MSCs are a niche representing 5% of pulp cells. They properties are similar to MSCs like a rapid proliferation, multilineage differentiation or growth factors secretion. DP-MSCs can differentiated into several types of cells such as adipocytes, osteocytes, chondrocytes, hepatocytes or skin cells [69]. Nakashima et al. [70] described two cases with DP-MSCs used. Stem cells were transplanted to root canals with granulocyte colony-stimulating factor in atelocollagen in 26- and 29-year old males. The source for DP-MSCs isolation was from removed third molar. Treatment results were a normal pulp, response to electrical testing and no periapical radiolucency [70]. Other clinical case studied the use of DP-MSCs isolated from deciduous tooth in 7 years old patient. Then, stem cells were applicated with lyophilized collagen-polyvinylpyrrolidone sponge to 61 years old patients with periodontal disease. After 3 and 6 months of transplantation, bone defect was volumetrically reduced, and the density of bone mineral was increased. No side effects such as a rejection or inflammatory reaction was observed [71]. Aimetti et al. present case series with DP-MSCs and collagen sponge used in patients with chronic periodontitis. A decrease in the depth of the lesion and an increase in the area of the attachment were noted. All periapical cavities were closed by the applied treatment, and the level of bone filling increased [72].

##### DF-MSCs

Dental follicle stem cells (DF-MSCs) are a unique population of cells in the dental follicle tissue. DF-MSCs take a part in formed a elements of periodontist, like a cementum, ligament or alveolar bone. They also differentiated to osteogenic, adipogenic and chondrogenic cells as well as ductal or salivary gland cells [73]. It is observed that DF-MSCs have a high proliferation capacity and anti-inflammatory properties. In inflammatory environment these cells are related to periodontal regeneration, through periostin release and macrophage reprogramming [74]. DF-MSCs are studied in animal models, Tian et al. [75] injected to dorsal skin in mice, DF-MSCs or periodontal ligament cells with dentin matrix scaffold. After 8 weeks the methods were compared. In total, 32 proteins with differentiated expression between groups were observed. Additionally, in DFS-MCs group the dentin tissue was more complex [75]. Huang et al. [76] used extracellular vesicles derived from DF-MSCs (DFC-sEV), which was pre-stimulated with lipopolysaccharide (LPS). Construct was administrated to dogs with induced periodontitis. Treatment method allowed to obtain antioxidant environment and can be used as a supportive therapy for patients with periodontal damage [76].

##### SHED

Stem cells from exfoliated deciduous teeth (SHED) are derived from cranial neural crest cells. They have the characteristics of MSCs. They show persistence of phenotypic traits during in vitro culture and cryopreserving. SHEDs can differentiated into cells from adipose tissue, cartilage, bone, smooth muscle or epithelium [77]. Clinical case of used SHEDs in periapical lesions was described by Prasad et al. 2017 [78]. The sources from stem cells were exfoliated deciduous teeth. After preparation of the dental canals, a construct consisting of a bioglass and SHEDs was used in regeneration of periapical lesion following root resection and removal of apical pathology. Closure of the periapical lesion was noted 30 days after therapy. No side effects of the treatment were observed [78].

##### Other Sources of MSCs

Other sources of mesenchymal stem cells can also be used in periodontal treatment. Bone marrow mesenchymal stromal cells (BM-MSCs) with stromal cell-derived factor 1 α (SDF-1α) and transglutaminase crosslinked gelatines were selected as a construct stimulated periodontal regeneration in in vitro studies. SDF-1 α enhance the migratory effect in stem cells and modulated macrophage polarization [79]. Nevertheless, Lemaitre et al. 2017 [80] used adipose-derived mesenchymal stromal cells (AD-MSCs) with collagen to periodontal damage in mouse model. Regeneration of the cementum and periodontal ligament was observed, as well as an increase in the number of vessels 12 weeks after the treatment, leading to the restoration of homeostasis in the tissues surrounding the tooth [80].

#### 4.2.3. Translational Challenges

It should be emphasized that despite promising preclinical data, there are currently no results of controlled clinical trials assessing the effectiveness of MSCs in the treatment of peri-implantitis in humans. Most available data comes from animal studies. However, survival, retention, engraftment, and vascularization remain largely untested in peri-implantitis models. Unfortunately, in the case of cell therapies, translating preclinical research results into the clinic often becomes a challenge.

One limitation is the cost of such therapy. Preparing cells for transplantation requires their isolation using appropriately purified enzymes and other reagents, as well as sterile conditions. Such procedures must be conducted in specialized GMP laboratories, where conditions are maintained to limit the possibility of cell product contamination. This all generates very high costs. Furthermore, if the procedure requires in vitro cell culture prior to transplantation, these costs increase. This makes such therapy expensive and often perceived as unprofitable [81,82].

Another problem is regulatory issues. Complicated registration procedures, both by the FDA and EMA, often prevent these cells from being routinely used in therapy. Furthermore, MSC therapy often uses cells of different origins, making it difficult to draw meaningful conclusions. There is also a lack of standardized procedures for cell preparation and administration [83,84].

Additionally, peri-implantitis is characterized by persistent biofilm and chronic inflammation. This conditions may compromise therapeutic efficacy of MSCs therapy. This hostile inflammatory environment remains translational challenge, despite the fact that peri-implantitis models demonstrated that administration of MSCs may reduce inflammatory mediators [60,63]. Results from periodontitis and periodontal regeneration studies suggest that chronic inflammation, dysregulated immune signaling and oxidative stress may impair MSCs retention, survival and regenerative activity or even induce apoptosis [85,86]. However, studies utilizing peri-implantitis models lack evaluation of long-term cell retention, survival, engraftment, stability of delivery, differentiation, regenerative function and vascular integration. This shows that there is still much research to be done as data is often extrapolated from other areas of regenerative medicine.

Another issue is biological diversity. Cells from different patients may have different properties and activities, which obviously translates into therapeutic effect. Using a patient’s own cells or allogeneic cells is a separate issue. There is currently no evidence to support the superior approach. Using allogeneic cells may be more practical, as it simplifies the preparation process. There is no need to isolate and culture cells separately for each patient. This also facilitates larger-scale cultivation [87,88]. Furthermore, such cells, for example, from a healthy donor, can be frozen and used when needed. Of course, the issue of cell cryopreservation carries potential drawbacks. The cryopreservation process itself can affect the quality of the cell preparation (e.g., by reducing viability). It is also necessary to have appropriate laboratory facilities for cell cryopreservation [89]. However, allogenic MSCs-based products do not eliminate biological heterogeneity. Donor age, tissue source, donor-to-donor variability, cell isolation and expansion procedures and batch-to-batch differences still constitute some limitation. They may influence cellular phenotype, secretory profile and therapeutic efficacy. Nevertheless, allogenic approaches may offer practical advantages through standardized manufacturing process, quality control, development of off-the-shelf therapies and broader treatment availability [90,91,92,93].

All these problems have led to a growing interest in cell-free solutions, such as EVs, in recent years [66].

## 5. Conclusions and Future Perspectives

Regenerative treatment of peri-implantitis remains clinically challenging because successful therapy requires simultaneous control of inflammation, effective implant-surface decontamination, stabilization of the regenerative compartment, and reconstruction of lost peri-implant bone. Current evidence indicates that reconstructive surgical procedures can improve radiographic defect fill and selected peri-implant clinical parameters; however, these benefits are not consistently accompanied by superior probing-depth reduction, inflammatory disease resolution, or other clinically relevant outcomes when compared with open-flap debridement alone [10]. No single regenerative biomaterial or reconstructive protocol has demonstrated universal clinical superiority across different peri-implantitis defects [25]. A recent meta-analysis similarly demonstrated significantly greater radiographic bone-level gain following regenerative treatment compared with open-flap debridement, while no significant advantages were identified for probing depth, bleeding on probing, suppuration, recession, or clinical attachment level [10].

Among currently evaluated materials, DBBM and porous titanium granules are supported by comparatively extensive direct clinical evidence, including randomized trials and long-term follow-up studies. Hydroxyapatite-based materials are also supported by randomized human data, although considerable heterogeneity in HA formulations and accompanying treatment protocols limits material-specific conclusions. BCP-containing reconstructive protocols have demonstrated favourable clinical and radiographic outcomes, including long-term maintenance in some cohorts, but BCP has rarely represented the randomized treatment variable itself. EMD and PRF may provide biologically active adjunctive effects; however, their independent contribution remains uncertain. In particular, a recent randomized clinical trial failed to demonstrate an incremental clinical, radiographic, composite, or patient-reported benefit when EMD was added to bone-graft and membrane-based reconstructive therapy [35].

A central limitation of the available literature is the discrepancy between radiographic defect reconstruction and complete clinical disease resolution. Residual graft particles or non-resorbable materials may contribute to the radiographic appearance of defect fill without demonstrating restoration of the original bone–implant interface. Consequently, future clinical studies should move beyond isolated radiographic endpoints and report a standardized core set of outcomes, including probing-depth reduction, bleeding and suppuration on probing, radiographic bone-level change or defect fill, implant survival, mucosal recession, treatment-related complications, patient-reported outcomes, and the need for retreatment or implant removal. Composite definitions of treatment success may provide additional clinical value, but their individual components should also be reported separately to allow meaningful comparison between studies [10].

Future randomized controlled trials should be multicentre and designed around a clearly prespecified primary clinical endpoint. Rather than adopting an arbitrary universal sample size, the required sample should be calculated a priori according to the expected clinically meaningful between-group difference, outcome variability, statistical power, and anticipated loss to follow-up. Standardized peri-implantitis case definitions, defect classifications, implant-surface characteristics, decontamination protocols, reconstructive procedures, maintenance programmes, and outcome definitions are essential to improve comparability between studies. Primary efficacy assessment should include at least 12 months of follow-up, while extended observation of 3–5 years or longer is desirable to evaluate recurrence, retreatment, implant survival, and the durability of reconstructive outcomes.

Patient and defect stratification should also become an integral component of future research. Treatment outcomes should be analysed according to defect morphology and containment, implant position and surface characteristics, smoking status, history of periodontitis, systemic risk factors, oral-hygiene control, peri-implant soft-tissue conditions, prosthetic accessibility, and adherence to supportive maintenance. Such stratification may help identify clinical situations in which reconstructive treatment is predictable and those in which alternative treatment strategies or implant removal may provide a more favourable prognosis [8,11].

In addition to conventional randomized trials, prospective multicentre registries may provide complementary real-world evidence regarding long-term effectiveness, complications, retreatment rates, implant survival, and treatment performance across different implant systems and patient populations. Registry-based studies may be particularly valuable for evaluating uncommon complications and long-term outcomes that are difficult to assess in conventional randomized trials.

Biologically enhanced biomaterials represent an important direction for future development. Examples already explored clinically include calcium-phosphate scaffolds modified with hyaluronic acid [57], hydroxyapatite-based materials combined with bioactive agents such as simvastatin [32], and autologous platelet-rich fibrin used alone or in combination with particulate regenerative materials [21,24]. These approaches illustrate a transition from primarily structural osteoconductive scaffolds toward multifunctional regenerative strategies designed to influence angiogenesis, inflammatory modulation, cellular recruitment, and bone formation. However, biological plausibility or favourable early outcomes should not be interpreted as evidence of clinical superiority until adequately powered comparative trials demonstrate durable patient-relevant benefits.

Emerging cell-based and cell-derived strategies may further expand the biological potential of regenerative therapy. However, the majority of currently available evidence relevant to peri-implant regeneration remains preclinical, translational, or extrapolated from periodontal and endodontic applications rather than derived from controlled human peri-implantitis trials. Their future clinical application will depend on demonstration of safety, reproducibility, standardized manufacturing, regulatory feasibility, cost-effectiveness, and clinically meaningful superiority over established regenerative protocols. Evidence derived from periodontal, endodontic, animal, or in vitro models should therefore remain clearly distinguished from direct human evidence in peri-implantitis.

Ultimately, regenerative treatment of peri-implantitis should not be regarded as a material-driven procedure. Biomaterial selection represents only one component of treatment success and must be integrated with effective disease control, appropriate defect selection, implant and prosthetic assessment, implant-surface decontamination, soft-tissue management, and long-term supportive maintenance [8,11,25]. Current evidence supports reconstructive therapy in appropriately selected cases, particularly in contained peri-implant defects, but the predictability of individual biomaterials remains limited by substantial clinical and methodological heterogeneity. Future progress is therefore more likely to arise from matching biological and structural regenerative strategies to specific defect characteristics and patient-related risk profiles than from identifying a single universally superior grafting material.

## Figures and Tables

**Table 1:** Comparative Summary of Direct Human Clinical Evidence for Regenerative Biomaterials Used in Peri-Implantitis Treatment.

Biomaterial/Strategy	Study	Design/Sample	Intervention and Comparator	Follow-Up	Main Clinical and Radiographic Outcomes	Main Limitations/Evidence Interpretation
Enamel matrix derivative (EMD)	Isehed et al. [26]	3- and 5-year follow-up of a randomized clinical trial; 29 patients originally randomized, 25 included in the survival analysis	Surgical treatment + EMD vs. surgical treatment without EMD	3 and 5 years	Implant survival at 3 years: 100% vs. 83%; at 5 years: 85% vs. 75% in EMD and control groups, respectively. EMD treatment and bone-level change were positively associated with implant survival.	Small sample and long-term follow-up of an existing RCT cohort. EMD was used as an adjunct; therefore, its independent regenerative effect remains uncertain.
Porous titanium granules (PTG)	Jepsen et al. [27]	Multinational, multicentre randomized controlled trial; 63 patients with one circumferential peri-implant intraosseous defect each	OFD + porous titanium granules (PTG) vs. OFD alone	12 months	Mean radiographic defect fill was 3.6/3.6 mm mesially/distally with PTG vs. 1.1/1.0 mm with OFD alone (*p* < 0.0001). Mean PPD reduction was 2.8 vs. 2.6 mm. BoP decreased from 89.4% to 33.3% with PTG and from 85.8% to 40.4% with OFD. Complete disease resolution was achieved in 30% vs. 23%, without a significant between-group difference.	Significant radiographic advantage, but no significant improvement in complete clinical disease resolution compared with OFD alone. Because PTGs are non-resorbable, radiographic defect fill cannot distinguish newly formed bone from retained titanium granules.
Porous titanium granules (PTG)	Guler et al. [28]	Prospective comparative clinical study; 22 patients with peri-implantitis defects	Rotary titanium brush + PTG + PRF membrane vs. xenograft + collagen membrane + PRF membrane	6 months	Mean CAL improved from 5.29 ± 1.06 to 3.59 ± 0.88 mm in the PTG group and from 4.77 ± 1.05 to 3.30 ± 0.58 mm in the xenograft group. Radiographic bone filling was significantly greater in the PTG group at 6 months.	Small sample and short follow-up. The treatment protocols differed in several components, including surface debridement and membrane/graft selection, preventing attribution of the observed differences to PTG alone.
DBBM + collagen membrane	Renvert et al. [29]	Multicentre randomized controlled trial; 66 patients	Surgical debridement + DBBM + native bilayer collagen membrane (NBCM) vs. surgical debridement alone	12 months	Radiographic defect fill at the deepest site was 2.7 ± 1.3 mm with DBBM + NBCM vs. 1.4 ± 1.2 mm with debridement alone (*p* < 0.0001). Mean PPD reduction was 1.9 vs. 2.3 mm, respectively (*p* = 0.578). No significant between-group differences were observed for BOP, suppuration, recession, or patient-reported outcomes. Using the study-defined composite endpoint (RDF ≥ 1.0 mm, PPD ≤ 5 mm, ≤1 of 4 sites with grade-1 BOP, and no suppuration), treatment success was achieved in 32% of the test group (*n* = 34) and 21% of the control group (*n* = 32).	DBBM + NBCM produced significantly greater radiographic defect fill, but no significant advantage in clinical inflammatory parameters, PPD reduction, or patient-reported outcomes. Because the intervention combined a bone substitute with a membrane, the independent contribution of DBBM cannot be determined.
DBBM with 10% collagen (DBBMC)	Roccuzzo et al. [30]	Prospective long-term cohort; 26 patients initially, 14 reached 10-year examination	Reconstructive surgery with DBBM containing 10% collagen (DBBMC); no untreated or alternative-treatment control group	10 years	Overall implant survival was 67% at 10 years, with 80% survival for SLA and 55% for TPS implants. Mean PD decreased from 6.6 ± 1.3 to 3.2 ± 0.7 mm in SLA implants and from 7.2 ± 1.5 to 3.4 ± 0.6 mm in TPS implants. Treatment success was achieved in 42% of SLA and 29% of TPS implants.	Long-term prospective data are clinically valuable, but substantial attrition, absence of a control group, additional retreatment during supportive care, and marked differences according to implant surface characteristics limit causal interpretation. The study demonstrates that long-term implant survival does not necessarily correspond to complete disease resolution.
DBBMC + EMD + doxycycline	Mercado et al. [31]	Prospective 3-year cohort study; 30 patients with peri-implantitis	Surgical debridement and implant-surface decontamination followed by DBBMC + EMD + doxycycline; no control group	36 months	Mean PPD decreased from 8.9 ± 1.9 to 3.50 ± 0.50 mm at 36 months, while mean radiographic bone loss decreased from 6.92 ± 1.26 to 2.60 ± 0.73 mm. The predefined study-specific successful treatment outcome was achieved in 56.6% of evaluable treated implants.	Prospective long-term clinical data, but no control group was included. DBBMC, EMD, and doxycycline were administered simultaneously, and additional soft-tissue grafting was performed when required; therefore, the contribution of any individual regenerative component cannot be determined.
Nanocrystalline HA + silica (NanoBone, ARTOSS GmbH, Rostock, Germany)	Soliman [32]	Prospective single-centre randomized controlled clinical trial; 30 patients, one peri-implantitis defect evaluated per patient	NanoBone vs. NanoBone + simvastatin	6 months	Mean PPD decreased from 6.20 ± 0.97 to 2.73 ± 0.77 mm with NanoBone alone and from 6.46 ± 1.02 to 2.80 ± 0.75 mm with NanoBone + simvastatin. The radiographic defect measure decreased from 4.05 ± 1.04 to 2.57 ± 1.45 mm vs. 3.86 ± 1.00 to 1.77 ± 0.70 mm, respectively, with significantly greater radiographic improvement in the simvastatin group.	Both groups received the same HA-based synthetic bone substitute; therefore, the trial primarily evaluated the adjunctive effect of simvastatin rather than the efficacy of HA-based grafting versus no graft. The sample was small and follow-up was limited to 6 months.
Enamel matrix derivative (EMD)	Isehed et al. [33]	Single-centre randomized controlled clinical trial; 26 patients with peri-implantitis	Open-flap debridement and implant-surface decontamination + EMD vs. the same surgical treatment without EMD	12 months	Median marginal bone-level change was +0.9 mm with EMD vs. −0.1 mm without EMD (*p* = 0.295); bone-level gain occurred in 75% vs. 46% of implants. Median PPD reduction was 2.8 vs. 3.0 mm (*p* = 0.270), with no significant between-group differences in clinical inflammatory outcomes. In multivariate analysis, adjunctive EMD was significantly associated with increased marginal bone level.	Small sample size. Although EMD was significantly associated with increased marginal bone level in multivariate modelling, univariate between-group differences in bone level, PPD, and inflammatory parameters were not statistically significant. Therefore, the study suggests a potential adjunctive radiographic/biological benefit but does not demonstrate consistent clinical superiority.
DBBM + native bilayer collagen membrane	Renvert et al. [34]	3-year follow-up of a multicentre randomized controlled trial; 50 patients completed the 36-month assessment	GBR with DBBM + native bilayer collagen membrane (NBCM) vs. open-flap debridement (OFD) alone	36 months	Mean radiographic defect fill was 2.13 ± 1.26 mm with DBBM + NBCM vs. 1.64 ± 1.54 mm with OFD alone (*p* = 0.18). No significant between-group differences were observed in PPD, BOP, suppuration, mucosal recession, or PROs. Using the study-defined composite endpoint (no additional bone loss, PPD ≤ 5 mm, no BOP, and no suppuration), treatment success was reported in 46.2% of the test group and 20.0% of controls (*p* = 0.053). Fifty patients completed the 36-month assessment, with 25 patients per group evaluable for the primary radiographic endpoint.	Long-term follow-up of an existing RCT rather than an independent cohort. Although numerically greater radiographic defect fill and composite treatment success were observed with GBR, between-group differences did not reach statistical significance at 36 months, and no significant advantage was detected for individual clinical parameters or PROs. Attrition further reduced the evaluable sample.
PRF	Hamzacebi et al. [24]	Randomized controlled clinical trial; 19 patients with peri-implant bone loss	Access flap surgery + PRF vs. access flap surgery alone	6 months	At 6 months, mean PPD reduction was 2.82 ± 1.03 mm with PRF vs. 2.05 ± 0.77 mm with access flap alone. Mean CAL gain was 3.31 ± 1.08 vs. 1.84 ± 0.81 mm, respectively. A significant increase in keratinized mucosa was also observed in the PRF group.	Very small sample and short follow-up. The study demonstrated favourable short-term clinical effects of adjunctive PRF, but did not provide long-term data on disease resolution, implant survival, radiographic reconstruction, or histological re-osseointegration.
Enamel matrix derivative (EMD)	Regidor et al., 2026 [35]	Randomized controlled trial; 40 patients, 44 implants	Bone graft + resorbable membrane + EMD vs. the same reconstructive protocol without EMD	12 months	PPD reduction: 4.3 ± 2.4 mm vs. 4.0 ± 1.7 mm (*p* = 0.105). No significant differences in clinical, radiographic, composite, or PRO outcomes.	Well-designed direct RCT but failed to demonstrate incremental benefit of EMD when added to graft- and membrane-based reconstruction.
Nanocrystalline HA (NHA)	Schwarz et al., 2009 [36]	4-year follow-up of randomized clinical cohort; 20 patients originally randomized	Access flap + NHA vs. access flap + natural bone mineral + collagen membrane	48 months	PPD reduction: 1.1 ± 0.3 vs. 2.5 ± 0.9 mm; CAL gain: 0.6 ± 0.5 vs. 2.0 ± 1.0 mm. Radiographic fill observed at 5 vs. 8 sites.	Groups differed in both graft material and membrane use, preventing a pure HA comparison. Long-term NHA outcome without membrane was considered poor.
Bovine-derived HA (EndoBon^®^)	Renvert et al., 2018 [37]	Randomized controlled trial; 41 patients with 3- or 4-wall defects	Surgical debridement + HA bone substitute vs. surgical debridement alone	12 months	Significant radiographic defect fill occurred only in the grafted group. Composite successful outcome: 42.9% vs. 5.0%; absence of BOP: 47.6% vs. 35.0%, without significant difference for BOP.	Strong direct comparison with debridement alone; radiographic/composite advantage did not translate consistently across inflammatory outcomes.
Bovine-derived HA (EndoBon^®^)	Polymeri et al., 2020 [38]	Randomized clinical pilot study; 24 patients	EndoBon^®^ vs. DBBM (Bio-Oss^®^)	12 months	Both groups improved significantly; mean intrabony defect-depth reduction approximately 2.5–3.0 mm. No significant between-group differences. Composite success: 0/13 HA vs. 2/11 DBBM (*p* = 0.199).	Small pilot sample; suggests comparable reconstructive performance but no superiority of HA over DBBM.
BCP (HA/β-TCP)	de Tapia et al., 2019 [39]	Double-blind randomized controlled trial; 30 patients	Both groups: HA/β-TCP + collagen membrane; titanium-brush decontamination vs. conventional mechanical/chemical decontamination	12 months	PPD reduction: 4.87 ± 1.55 vs. 2.85 ± 1.91 mm (*p* = 0.009); residual PPD: 3.60 ± 0.91 vs. 4.92 ± 1.26 mm (*p* = 0.007).	Direct peri-implantitis study of a BCP-containing protocol, but BCP was used in both groups; therefore, the trial tests the titanium brush, not BCP itself.

Note: Definitions of treatment success and disease resolution differed between studies and included different combinations of probing depth, bleeding or suppuration on probing, radiographic bone-level stability or defect fill, and implant survival. Accordingly, reported success percentages should be interpreted according to the study-specific outcome definition and evaluable population and should not be directly compared across studies. Long-term follow-up publications derived from previously reported clinical cohorts are presented separately to illustrate outcome durability and should not be interpreted as independent study populations.

**Table 2:** Comparative Biological, Structural, and Clinical Characteristics of Regenerative Biomaterials Used in Peri-Implantitis Treatment.

Material/Strategy	Primary Biological Role	Resorption/Persistence	Space-Maintaining Capacity	Direct Clinical Evidence in Peri-Implantitis	Main Clinical Advantage	Main Limitation	Relative Cost/Resource Burden *
Enamel matrix derivative (EMD)	Bioactive modulation of wound healing, cellular activity and inflammatory response	Biologically degraded; no persistent scaffold	Low	RCT and long-term clinical follow-up available [26,33,35]	Biologically active adjunct with potential influence on healing and inflammatory modulation	No structural support; independent contribution difficult to separate from accompanying surgery and grafting procedures	High
Porous titanium granules (PTG)	Osteoconductive scaffold and mechanical stabilization of the defect	Non-resorbable; permanent	High	RCTs and prospective comparative studies available [27,28,40,41]	Excellent structural stability and maintenance of defect volume; increased radiographic defect fill compared with OFD alone	Remains permanently within the reconstructed site; radiographic fill may partly represent retained titanium rather than newly formed bone	High
Deproteinized bovine bone mineral (DBBM)	Osteoconductive scaffold supporting vascular and bone ingrowth	Very slow/limited resorption; long-term persistence	Moderate–High	RCTs, prospective studies, and long-term follow-up of up to 7–10 years [29,30,31,34,42]	Extensive clinical experience, good volume stability, and comparatively strong long-term peri-implantitis evidence	Residual graft particles may persist for years; disease resolution remains variable and superiority over other materials is not established	Moderate
Hydroxyapatite (HA)	Osteoconductive mineral scaffold	Variable, generally slow, depending on crystallinity, porosity and formulation	Moderate	Randomized and comparative human peri-implantitis studies available, with heterogeneous HA formulations [32,36,37,38,43]	High biocompatibility and similarity to the mineral phase of bone	Substantial heterogeneity among HA formulations and accompanying protocols; persistent particles may complicate radiographic interpretation; superiority over alternative materials is not established.	Low–Moderate
Biphasic calcium phosphate (BCP; HA/β-TCP)	Osteoconductive scaffold with adjustable degradation profile	Variable; β-TCP resorbs faster, whereas HA provides longer-term persistence	Moderate	Human peri-implantitis evidence is available for BCP-containing reconstructive protocols, but material-specific evidence remains limited [39,44]	Synthetic material with tunable resorption behaviour and no xenogeneic origin	Considerable heterogeneity between HA/β-TCP ratios and formulations; limited direct evidence for established peri-implantitis	Low–Moderate
Platelet-rich fibrin (PRF)	Autologous biological enhancement of angiogenesis, cellular recruitment and soft-tissue healing	Rapid/short-term biological degradation	Low	Direct controlled peri-implantitis evidence remains limited [24]	Autologous source of fibrin matrix, platelets, leukocytes and growth factors; may improve early healing and graft handling	Limited mechanical stability; cannot replace a conventional barrier membrane or rigid space-maintaining device	Low–Moderate

* Relative cost/resource burden is presented qualitatively and may vary substantially according to country, manufacturer, product formulation, and clinical protocol. Direct comparative cost-effectiveness studies between these regenerative strategies are limited; therefore, these categories should not be interpreted as formal economic evidence.

## Data Availability

No new data were created or analyzed in this study. Data sharing is not applicable to this article.

## Supplementary Materials

The following supporting information can be downloaded at: https://www.mdpi.com/article/10.3390/jfb17100505/s1. Supplementary File S1: Database-Specific Search Strategies.

## Abbreviations

A-PRF	advanced platelet-rich fibrin
AD-MSCs	adipose-derived mesenchymal stromal cells
BCP	biphasic calcium phosphate
BM-MSCs	bone marrow-derived mesenchymal stromal cells
BMP	bone morphogenetic protein
CAL	clinical attachment level
CBCT	cone-beam computed tomography
DBBM	deproteinized bovine bone mineral
DBBM-C	deproteinized bovine bone mineral with collagen
DBBMC	deproteinized bovine bone mineral with 10% collagen
DFC-sEVs	dental follicle cell-derived small extracellular vesicles
DF-MSCs	dental follicle mesenchymal stromal cells
DP-MSCs	dental pulp mesenchymal stromal cells
EMD	enamel matrix derivative
GBR	guided bone regeneration
GMSCs	gingival mesenchymal stem cells
GTR	guided tissue regeneration
HA	hydroxyapatite
HAP	hydroxyapatite
I-PRF	injectable platelet-rich fibrin
ISQ	implant stability quotient
LPS	lipopolysaccharide
LRAP	leucine-rich amelogenin peptide
MSC	mesenchymal stromal cell
mSBI	modified sulcus bleeding index
NBCM	native bilayer collagen membrane
OFD	open-flap debridement
PDGF	platelet-derived growth factor
PI	plaque index
PPD	probing pocket depth
PRF	platelet-rich fibrin
PTG	porous titanium granules
RCF	relative centrifugal force
SCAPs	stem cells from the apical papilla
SCDI	stability coefficient of dental implants
SDF-1α	stromal cell-derived factor 1 alpha
SHED	stem cells from human exfoliated deciduous teeth
SLA	sandblasted, large-grit, acid-etched
TCP	tricalcium phosphate
TGF-β	transforming growth factor beta
TPS	titanium plasma-sprayed
VEGF	vascular endothelial growth factor
β-TCP	beta-tricalcium phosphate
