# Supplementary material for: Regenerative Biomaterials and Emerging Biological Strategies for Peri-Implantitis: Current Clinical Evidence from a Structured Narrative Review
Source: J Funct Biomater. 2026 Oct 6;17(10):505. doi: 10.3390/jfb17100505 (PMC13705906; doi:10.3390/jfb17100505)
Supplement: Supplementary file 1 [file jfb-17-00505-s001.zip › jfb-4495653-supplementary.pdf]

Supplementary File S1. Database-Specific Search Strategies

PubMed/MEDLINE

("peri-implantitis" OR periimplantitis OR "peri implantitis") AND (regeneration OR regenerative OR "guided bone regeneration" OR biomaterial OR biomaterials OR "bone graft" OR "bone grafts" OR "bone substitute" OR "bone substitutes" OR "deproteinized bovine bone mineral" OR DBBM OR hydroxyapatite OR "biphasic calcium phosphate" OR "tricalcium phosphate" OR "titanium granules" OR "porous titanium granules" OR "enamel matrix derivative" OR Emdogain OR "platelet-rich fibrin" OR PRF OR "mesenchymal stromal cells" OR "mesenchymal stem cells" OR "dental stem cells" OR "cell-based therapy" OR "extracellular vesicles" OR secretome)

Scopus

TITLE-ABS-KEY (("peri-implantitis" OR periimplantitis OR "peri implantitis") AND (regenerat* OR "guided bone regeneration" OR biomaterial* OR "bone graft*" OR "bone substitute*" OR "deproteinized bovine bone mineral" OR DBBM OR hydroxyapatite OR "biphasic calcium phosphate" OR "tricalcium phosphate" OR "titanium granule*" OR "porous titanium granule*" OR "enamel matrix derivative" OR Emdogain OR "platelet-rich fibrin" OR PRF OR "mesenchymal stromal cell*" OR "mesenchymal stem cell*" OR "dental stem cell*" OR "cell-based therap*" OR "extracellular vesicle*" OR secretome))

Web of Science Core Collection

TS=(("peri-implantitis" OR periimplantitis OR "peri implantitis") AND (regenerat* OR "guided bone regeneration" OR biomaterial* OR "bone graft*" OR "bone substitute*" OR "deproteinized bovine bone mineral" OR DBBM OR hydroxyapatite OR "biphasic calcium phosphate" OR "tricalcium phosphate" OR "titanium granule*" OR "porous titanium granule*" OR "enamel matrix derivative" OR Emdogain OR "platelet-rich fibrin" OR PRF OR "mesenchymal stromal cell*" OR "mesenchymal stem cell*" OR "dental stem cell*" OR "cell-based therap*" OR "extracellular vesicle*" OR secretome))
